# An improvement in the method of correcting indirect radial strain measurements during triaxial strength tests in rocks

**DOI:** 10.1016/j.mex.2024.102918

**Published:** 2024-08-15

**Authors:** E. Martínez-Bautista, D. Ibarra-González, J. Arzúa, M.A. González-Fernández, L.R. Alejano

**Affiliations:** a Department of Metallurgical and Mining Engineering, Universidad Católica del Norte, Chile; b CINTECX. GESSMin Group. Department of Natural Resources and Environmental Engineering, Universidade de Vigo, Spain

**Keywords:** Rock testing, Compressive strength test, Radial strain, Improved Indirect Radial Strain Correction for Triaxial Tests

## Abstract

The present article provides an improvement in the method to correct indirect strain measurements in triaxial compressive strength tests through axial displacement and hydraulic fluid volume change measurements. The improvement focused on reducing the parameters of the formula proposed for indirect volumetric strain in the original method, thereby facilitating the development of a simpler formula in which the radial strain depends on only two parameters: the initial volume of the rock specimen and the volume changes of the hydraulic fluid for each instant. The comparison between the improvement proposed, and original method resulted in a mean absolute difference of 0.003.

• This improvement does not depend on the axial strain, unlike the original method, which requires correcting the indirect axial strain measurements before correcting the indirect radial strain measurements.
• This improvement can be useful for research on the stress-strain behavior of intact rock under laboratory conditions, such as in the study of the post-peak state.

## Graphical abstract

**Figure:**
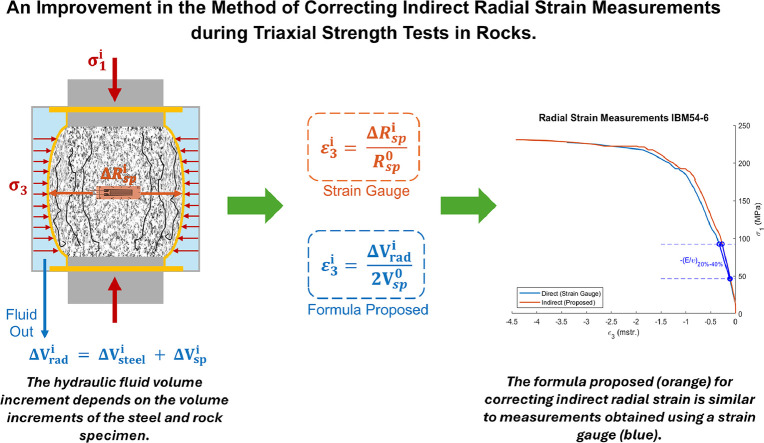

Specifications table

**Table:** 

Subject area:	Earth and Planetary Sciences
More specific subject area:	Rock Mechanics – Laboratory.
Name of your method:	Improved Indirect Radial Strain Correction for Triaxial Tests.
Name and reference of original method:	Alejano, L. R., Estévez-Ventosa, X., González-Fernández, M. A., Walton, G., West, I. G., González-Molano, N. A., & Alvarellos, J. (2021). A Method to Correct Indirect Strain Measurements in Laboratory Uniaxial and Triaxial Compressive Strength Tests. Rock Mechanics and Rock Engineering, 54(6), 2643–2670. https://doi.org/10.1007/s00603-021-02392-4 ISRM (2007). The complete ISRM suggested methods for rock characterization, testing and monitoring; 1974−2006. Suggested Methods for Determining the Uniaxial Compressive Strength and Deformability of Rock Materials Compressive Strength and Deformability of Rock Materials, 628.
Resource availability:	There are no special resources.

## Method details

The mechanical behavior of intact rock materials has been widely studied, particularly under laboratory conditions. This behavior can be studied through uniaxial and triaxial compressive strength tests to determine Young's modulus and Poisson's ratio, which are considered the main deformability properties of the intact rocks [1].

According to the methods suggested by the International Society for Rock Mechanics and Rock Engineering (ISRM) [2], there are two instruments to measure strain: strain gauges that obtain direct strain measurements, and Linear Variable Differential Transducers (LVDT) that obtain indirect strain measurements. However, there is no clarity on the appropriate use of these instruments. It is important to highlight that a significant difference is observed between the strain gauge and LVDT measurements [3,4]. This contrast can be attributed to strain phenomena associated with the different interfaces existing on the test setup. It is noteworthy that the strain measured by the LVDTs is significantly greater than that obtained using strain gauges [5,6]. Considering these observations, a distinct requirement emerges for a methodology that ensures the dependable application of corrections to the indirect strain measurements.

Early correction of indirect volumetric strain was documented by Farmer [7], who proposed a formula to determine the volumetric strain ($\varepsilon_{v}$) during triaxial compressive strength tests using both the hydraulic fluid volume changes measured in Hoek's cell and the axial displacement measured by LVDT (Eq. (1)). However, this method only considers the Young's modulus of steel to consider its deformation behavior.

(1) $$\begin{array}{c} \varepsilon_{v}=\frac{▵V}{V}\left(\%\right)=\frac{100}{V}\left[fV_{0}-\left(\pi r^{2}-\frac{F}{E}\right)l\right] \end{array}$$

where ΔV and V are the volume increment and initial volume of the rock specimen, respectively, f is the compressibility factor of the hydraulic fluid, $V_{0}$ is the volume of hydraulic fluid displaced, r is the radius of Hoek's cell steel bases or rams, F is the axial force, E is the Young's modulus of steel and l is the axial displacement.

Subsequently, Alejano et al. [3] proposed a method for correcting indirect strain measurements. This method is based on an energy approach to correct the axial strain measured indirectly by LVDT and volume changes of the hydraulic fluid measured during the triaxial compressive strength test (Eq. (2)).

(2) $$\varepsilon_{v}=\frac{▵V}{V}=\frac{\Delta V_{\text{rad}}-\pi {\left(r_{\text{steel}}^{0}\right)}^{2}h_{\text{sp}}^{0}\frac{\varepsilon_{1}^{i}}{1000}-\text{infinit}.}{V_{\text{sp}}^{0}}$$

where $\Delta V_{\text{rad}}$ is the radial (or lateral) volume increment, $r_{\text{steel}}^{0}$ is the initial radius of the steel bases, $h_{\text{sp}}^{0}$ is the initial height of the rock specimen, $\varepsilon_{1}^{i}$ is the corrected axial strain at each instant (in mstr.), $V_{\text{sp}}^{0}$ is the initial volume of the rock specimen, and there is an infinitesimal term, that can be disregarded.

The present article proposes an improvement in the calculation of indirect volumetric strain measurements proposed by Alejano et al. [3]. The proposed improvement was developed from the triaxial test results of 71 intact rock specimens of Blanco Mera granite, a moderately researched granite [[8], [9], [10], [11], [12], [13]], at different confinements of 0.2, 2.5, 5, 7.5, 10, 12.5, and 15 MPa and diameters of 38, 54, and 84 mm.

It was assumed that the axial stress magnitudes applied to the rock specimens were not sufficiently high to produce significant changes in the radius of the steel bases for all instants of the triaxial compressive strength tests, also this radius is very similar to the rock specimen radius (Eq. (3)).

(3) $$r_{\text{steel}}^{i}={\;r}_{\text{steel}}^{0}\left(1+\frac{\upsilon_{\text{steel}}}{E_{\text{steel}}}\sigma_{1}^{i}\right)\approx {\;r}_{\text{steel}}^{0}=r_{\text{sp}}^{0}$$

where $r_{\text{steel}}^{i}$ is the radius of the steel bases for each instant, $r_{\text{sp}}^{0}$ is the initial radius of the rock specimen, $\upsilon_{\text{steel}}$ is the Poisson's ratio of steel, $E_{\text{steel}}$ is the Young's modulus of steel, and $\sigma_{1}^{i}$ is the axial stress at each instant.

According to the above, the initial volume of the steel bases ($V_{\text{steel}}^{0}$), the volume at each instant ($V_{\text{steel}}^{i}$), and the volume change at each instant (${\Delta V}_{\text{steel}}^{i}$) of the steel bases inside the Hoek's cell can be considered as:

(4) $$V_{\text{steel}}^{0}=\pi {\left(r_{\text{steel}}^{0}\right)}^{2}h_{\text{steel}}^{0}$$

(5) $$V_{\text{steel}}^{i}=\;\pi {\left(r_{\text{sp}}^{0}\right)}^{2}\left(h_{\text{sleeve}}-{\;h}_{\text{sp}}^{i}\right)$$

(6) $${\Delta V}_{\text{steel}}^{i}=-\pi {\left(r_{\text{sp}}^{0}\right)}^{2}{\Delta h}_{\text{sp}}^{i}$$

where $h_{\text{steel}}^{0}$ is the initial distance between lower and upper platen, $h_{\text{sleeve}}$ is the height of the plastic sleeve, and ${\Delta h}_{\text{sp}}^{i}$, $h_{\text{sp}}^{i}$ are the height increment and height of the rock specimen at each instant, respectively.

On the other hand, the volume increment of the rock specimen at each instant (${\Delta V}_{\text{sp}}^{i}$) depends on the height and radius change of the rock specimen (${\Delta h}_{\text{sp}}^{i}$ and ${\Delta r}_{\text{sp}}^{i}$, respectively) during the triaxial compressive strength test, such that:

(7) $$\varepsilon_{V}^{i}=\frac{{\Delta V}_{\text{sp}}^{i}}{V_{\text{sp}}^{0}}=\varepsilon_{1}^{i}+2\varepsilon_{3}^{i}\Rightarrow {\Delta V}_{\text{sp}}^{i}=V_{\text{sp}}^{0}\varepsilon_{1}^{i}+2V_{\text{sp}}^{0}\varepsilon_{3}^{i}=\;\pi {\left(r_{\text{sp}}^{0}\right)}^{2}{\Delta h}_{\text{sp}}^{i}+2{\pi r}_{\text{sp}}^{0}h_{\text{sp}}^{0}{\Delta r}_{\text{sp}}^{i}$$

where $\varepsilon_{V}^{i}$ is the volumetric strain, $\varepsilon_{1}^{i}$ is the axial strain, and $\varepsilon_{3}^{i}$ is the radial strain of the rock specimen at each instant.

Therefore, the volume increment of the hydraulic fluid at each instant (${\Delta V}_{\text{rad}}^{i}$) for a triaxial compressive strength test can be expressed as:

(8) $${\Delta V}_{\text{rad}}^{i}={\Delta V}_{\text{sp}}^{i}+{\Delta V}_{\text{steel}}^{i}=2{\pi r}_{\text{sp}}^{0}h_{\text{sp}}^{0}{\Delta r}_{\text{sp}}^{i}$$

Finally, the radial and volumetric strains of a rock specimen at each instant during a triaxial strength test can be determined indirectly (Eq. (9),(10)).

(9) $$\varepsilon_{3}^{i}=\frac{{\Delta r}_{\text{sp}}^{i}}{r_{\text{sp}}^{0}}=\frac{{\Delta V}_{\text{rad}}^{i}}{2V_{\text{sp}}^{0}}$$

(10) $$\varepsilon_{v}^{i}=\frac{{\Delta V}_{\text{rad}}^{i}+V_{\text{sp}}^{0}\varepsilon_{1}^{i}}{V_{\text{sp}}^{0}}$$

The proposed improvement for the method proposed by Alejano et al. [3] is simpler, and the radial strain depends only on the initial volume of the rock specimen and the volume change of the hydraulic fluid at each instant, unlike the methods of Farmer [7] and Alejano et al. [3].

**Fig. 1:**
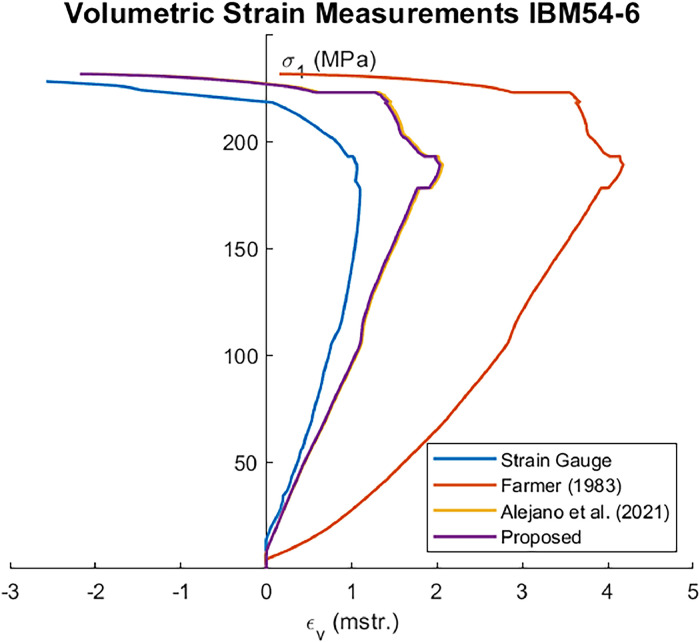
Volumetric stress-strain curves were obtained from strain gauges and the different approaches of a triaxial strength test, please note that the method of Alejano et al. [3] and the proposed method mostly overlap.

Corrections made using the Farmer's equation (Eq. (1)) were less precise than those of the other two approaches, whereas the current improvement did not show significant differences compared to the method proposed by Alejano et al. [3] (Fig. 1).

**Fig. 2:**
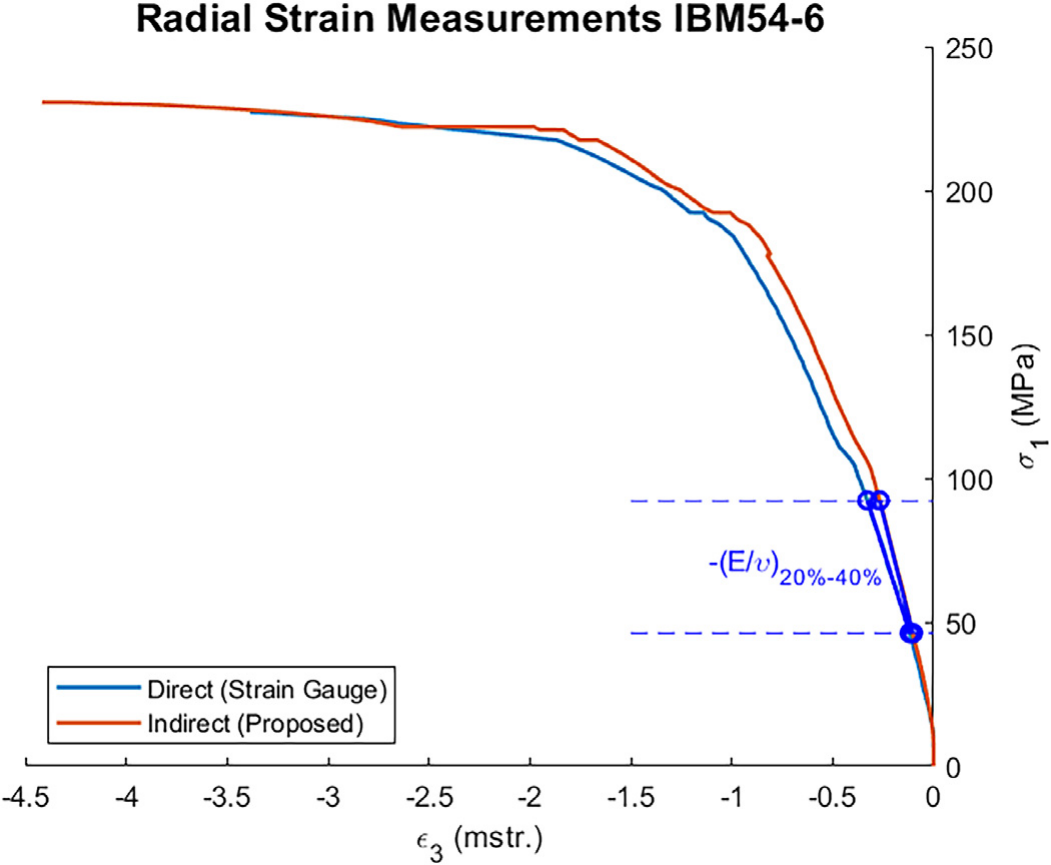
Direct (strain gauges) and indirect (using the proposed approach) axial stress-radial strain curves of a triaxial strength test.

The proposed improvement also yielded radial strain measurements comparable to those obtained using strain gauges (Fig. 2).

**Fig. 3:**
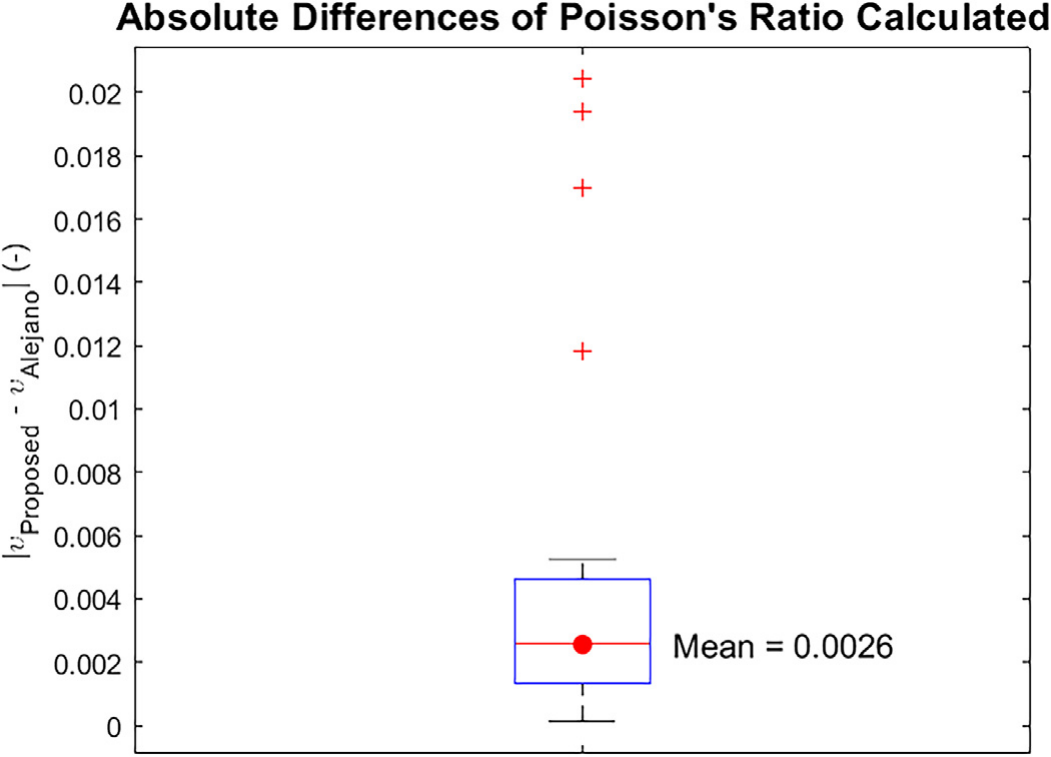
Absolute differences of Poisson's ratio of tests.

The Poisson's ratios calculated in the range of 20 % to 40 % of the maximum strength [12] of each test (Fig. 2) were calculated using the proposed approach and the original method, as shown in Table 1. The mean absolute difference (without outliers) between the proposed improvement and the original method is 0.0026 (Fig. 3).

**Table 1:** Mean Poisson's ratios at each diameter and confinement.

Diameter (mm)	Confinement (MPa)	Mean Poisson's Ratio (-)	
		Proposed Approach	Alejano et al. (2021)
38	0.2	0.148	0.122
38	2.5	0.227	0.227
38	5.0	0.240	0.241
38	7.5	0.250	0.360
38	10.0	0.177	0.176
38	12.5	0.266	0.266
38	15.0	0.126	0.128
54	0.2	0.296	0.292
54	2.5	0.181	0.177
54	5.0	0.171	0.166
54	7.5	NaN	0.217
54	10.0	0.186	0.182
54	12.5	0.418	0.415
54	15.0	0.164	0.161
84	0.2	0.261	0.275
84	2.5	NaN	0.181
84	5.0	0.404	0.420
84	7.5	0.108	0.120
84	10.0	0.229	0.260
84	12.5	NaN	NaN
84	15.0	NaN	0.231

It is important to note that outliers were excluded using the interquartile range (IQR) method to calculate the mean (Fig. 3). Although there is a possibility that variations in test execution or rock characteristics could have influenced the results, the overall trend of the data justifies the exclusion of these outliers. Moreover, the proposed approach is specifically applicable to triaxial compressive strength tests that use water as the hydraulic fluid, as it is an incompressible fluid [2]. However, this approach has only been validated on Blanco Mera granite. Therefore, further research would be beneficial to ascertain the applicability and universalization of this approach to all rock types.

## Declaration of generative AI and AI-assisted technologies in the writing process

During the preparation of this work the author(s) used Paperpal in order to improve writing. After using this tool/service, the author(s) reviewed and edited the content as needed and take(s) full responsibility for the content of the publication.

## CRediT authorship contribution statement

**E. Martínez-Bautista:** Conceptualization, Data curation, Writing – original draft. **D. Ibarra-González:** Data curation, Writing – original draft. **J. Arzúa:** Supervision, Funding acquisition. **M.A. González-Fernández:** Methodology, Supervision. **L.R. Alejano:** Resources, Methodology, Supervision.

## Declaration of competing interest

The authors declare that they have no known competing financial interests or personal relationships that could have appeared to influence the work reported in this paper.

## Data Availability

The authors do not have permission to share data.
